# Analysis of the p53 gene and papillomavirus detection in smears from cervical lesions

**DOI:** 10.1590/S1516-31802002000100006

**Published:** 2002-01-02

**Authors:** Ledy do Horto dos Santos Oliveira, André de Paula Fernandez, Brunno Lessa Saldanha Xavier, Eliana de Vasconcelos Machado Rodrigues, Silvia Maria Baeta Cavalcanti

**Keywords:** HPV, p53, Cervical lesions, HPV, p53, Lesões cervicais

## Abstract

**CONTEXT:**

Alterations of the p53 tumor suppressor gene are correlated with a critical step in the development of many human cancers. The tumor suppressor gene functions include regulation of the cell cycle and the cellular response to DNA damage, initiation of DNA repair and replication, induction of apoptosis and promotion of cell differentiation.

**CASE REPORT:**

Smears from ten cases of cervical lesions were analyzed for status of exons 5-8 of the p53 gene using PCR/SSCP. HPV infection was also screened by the PCR method using two PCR primer sets. Changes in the p53 gene were observed in a case of squamous carcinoma and a case of asymptomatic cervical intraepithelial neoplasia grade III (CIN III). High-risk HPV was detected in both cases showing that HPV infection and p53 mutation are not exclusive events.

## RESUMO

**CONTEXTO:**

Alterações no gene supressor de tumor p53 estão associadas a uma fase crítica no desenvolvimento de muitos canceres humanos. As funções desse gene incluem a regulação do ciclo celular e da resposta a danos no DNA, iniciação do reparo e da replicação do DNA, indução da apoptose e promoção da diferenciação celular. A inativação da p53 é o resultado de vários eventos incluindo mutações no gene p53 (com ou sem deleções alélicas associadas), ligação da p53 a proteínas virais ou celulares, e apreensão citoplasmática da proteína.

**RELATO DE CASO:**

Esfregaços de 10 pacientes com lesões cervicais foram analisados para verificação de mutações nos exons 5 a 8 do gene para a p53 por PCR/SSCP. Infecção por HPV foi também investigada por PCR utilizando-se dois conjuntos de oligonucleotídeos marcadores. Alterações no gene para p53 foram observadas em um caso de carcinoma escamoso e um de neoplasia epitelial cervical grau III (NIC III). Tipos de HPV de alto risco foram detectados em ambos os casos, confirmando que infecção por HPV e mutações na p53 não são eventos excludentes.

## INTRODUCTION

Alterations of the p53 tumor suppressor gene are correlated with a critical step in the development of many human cancers. The tumor suppressor gene functions include regulation of the cell cycle and the cellular response to DNA damage, initiation of DNA repair and replication, induction of apoptosis and promotion of cell differentiation. Inactivation of p53 may result from a number of events including mutation of the p53 gene, binding of p53 to cellular or viral proteins and cytoplasm sequestration of the protein.^1^ In cervical carcinoma, loss of p53 function can occur through its interaction with the E6 protein of oncogenic HPV types. In addition, DNA of these HPV types is often found integrated into cellular DNA. This integration can result in deletion or mutation of some viral genes.^2^

The clinical significance of p53 changes has been evaluated elsewhere for a wide variety of human cancers, including cervical cancer.^3^ Although cervical low-grade neoplasia can progress to cervical cancer, few molecular studies have indicated p53 mutation at this stage of the lesion.

In the present study, we investigate possible mutations in the region of exons 5-8 of the p53 gene and HPV infection in women who underwent routine Papa-nicolaou testing.

## METHODS

This study concerns ten women who were attended to at the Maternal-Child Department of the Medical School of the Universidade Federal Fluminense, Rio de Janeiro, between April and December 2000. Colpocytology test screening was performed at the first visit to the clinic. Biopsies were performed for women with abnormal cervical cytology. All patients gave written informed consent. The cases were histologically classified as ASCUS/CIN 0 (atypical squamous cells of undetermined significance), condyloma, low-grade squamous intraepithelial lesions (CIN I), high-grade squamous intraepithelial lesions (CIN II and III) and squamous invasive carcinoma.

HPV DNA was detected by using the MY09/11 consensus primer, which amplifies 460 bp DNA sequences within the L1 region. β-globin primers (0.1 pmol each), which amplify a 280 bp region of human DNA, were used as internal controls. HPV typing was done by PCR amplification with primers from the E6 gene DNA sequences of HPV 6, 11, 16, 18, 31, 33, and 35. PCR products were analyzed on 1.3% agar gel with ethidium bromide staining.^2^

PCR amplification of p53 exons 5 to 8 was carried out for SSCP analysis as previously described by Pinheiro et al. (1999).^4^

## RESULTS

The results are summarized in the Table. Abnormal bands were detected in two cases: one squamous carcinoma with parametrium involvement (exon 8) and one asymptomatic CIN III (exon 7) (Figure). The case diagnosed as squamous neoplasia was negative to L1 HPV primers, but it was positive to HPV 16 and 18 E6 gene. The CIN III case was also infected with both HPV 16 and 18.

**Table:** Clinical and cytological/histological findings associated with HPV detection and p53 status

*Patient*	*Clinical finding*	*Cytology/histology*	*HPV detection*		*P53 results*	
			*L1*	*type/E6*	*Status*	*exon*
*1*	*Asymptomatic*	*CIN II*	*-*	N**	*N/D*	
*2*	*Asymptomatic*	*CIN III*	*+*	*16*	*N/D*	
*3*	*Invasive lesion*	SC*	*-*	*16, 18*	*Mutant*	*8*
*4*	*Asymptomatic*	*CIN II/HPV*	*-*	*N*	*N/D*	
*5*	*Condyloma*	*ASCUS/HPV*	*+*	*18*	*N/D*	
*6*	*HIV*	*CIN II/HPV*	*+*	*6*	*N/D*	
*7*	*Condyloma*	*CIN I/HPV*	*+*	*6*	*N/D*	
*8*	*Asymptomatic*	*CIN I/HPV*	*+*	*6*	*N/D*	
*9*	*Asymptomatic*	*ASCUS*	*+*	*6*	*N/D*	
*10*	*Asymptomatic*	*CIN III*	*+*	*16, 18*	*Mutant*	*7*

* *Invasive squamous carcinoma;*

** *No HPV detected; N/D Gene mutations analyzed were not detected.*

**Figure:**
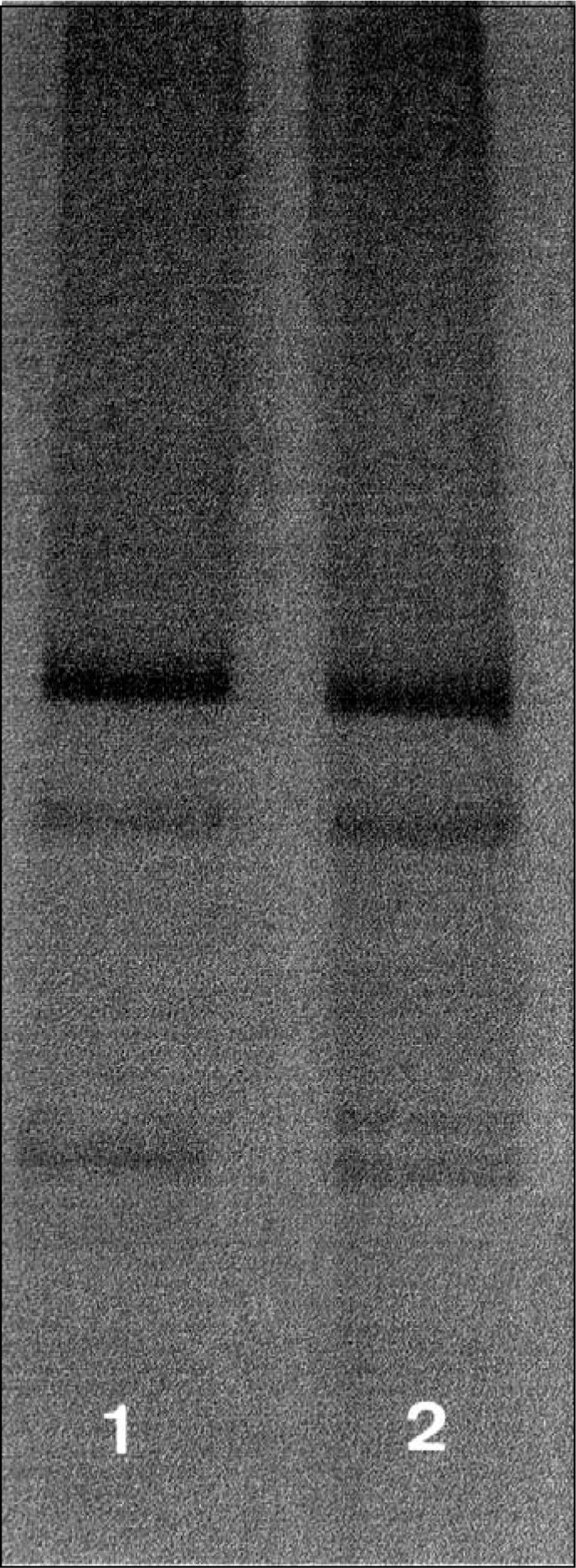
Screening of p53 mutations on exon 7 by PCR/SSCP. No. 1: normal run. No. 2: altered DNA (CIN III).

We found HPV type 6 (low risk) in the CIN III lesion of an HIV seropositive woman. The patient was negative for the oncogenic HPV types analyzed in this paper.

## DISCUSSION

The status of the p53 gene and HPV infection were analyzed in ten cervical lesions. Point mutations were detected in two patients with premalignant and malignant lesion, respectively, by PCR/SSCP. No p53 mutation was found in benign lesions, in agreement with the literature^3^. The PCR/SSCP method has high sensitivity and specificity for the detection of mutations comparable to DNA sequencing.^4^

P53 alterations are not frequent in cervical cancer, being generally detected in negative HPV tumors.^5^ However, we found two cases simultaneously positive for p53 mutation and high-risk HPV infection. One of the patients presented an invasive carcinoma and the other presented CIN III. Although other authors have reported p53 mutation as occurring only in a late stage of this disease,^3^ it is worth noting that we found a p53 mutation in one premalignant case, suggesting that it may have occurred early and thus may contribute to the process of cancer establishment.

We detected HPV in eight of the ten cases by using two sets of HPV primers. Differences in HPV positivity were found in squamous carcinoma, negative to L1 gene but positive for type-specific primers. Monk et al.^6^ have already described the same findings. We suggest that the L1 gene may have been deleted during the integration of HPV DNA. Hence, the use of two sets of primers may increase HPV detection in cervical tumors, elucidating the etiology of these cancers.

In conclusion, although we only studied a small number of cervical lesions, we have shown that p53 mutations and oncogenic HPV infection are not mutually exclusive events and thus may cooperate in the establishment of malignant cells.
